# Chemical Imaging of Platinum-Based Drugs and their Metabolites

**DOI:** 10.1038/srep38507

**Published:** 2016-12-05

**Authors:** Xin Liu, Amanda B. Hummon

**Affiliations:** 1 Department of Chemistry and Biochemistry Harper Cancer Research Institute University of Notre Dame McCourtney Hall Notre Dame, IN 46556, USA

## Abstract

Platinum-based drugs (cisplatin, carboplatin, and oxaliplatin) are widely used therapeutic agents for cancer treatment. Even though the platinum (Pt)-drugs are routinely used clinically, a clear picture of their distribution within tumor tissues is lacking. The current methods to image the distribution of Pt drugs are limited and do not enable the discrimination of the drug from its metabolites. In this manuscript, we demonstrate a methodology that enables chemical imaging of a Pt drug and its metabolites simultaneously and specifically. Matrix-Assisted Laser Desorption/Ionization (MALDI) Mass Spectrometry Imaging (MSI) is combined with an on-tissue chemical derivatization using diethyldithiocarbamate (DDTC). DDTC abstracts the Pt atom to generate ionizable complexes that can be imaged by MALDI MSI. We demonstrate that Pt drugs and their metabolites can be specifically imaged. This approach was successfully applied to map the penetration and metabolism of oxaliplatin in hyperthermic intraperitoneal chemotherapy (HIPEC)-like treated 3D colorectal tumor mimics. The distribution of cisplatin and carboplatin was mapped in additional 3D tumor mimics. We demonstrate that the approach can also be used to image the distribution of copper ions in cells. This method has the potential to be used to evaluate the penetration and distribution of a wide range of compounds.

Platinum (Pt) drugs, such as cisplatin, carboplatin, and oxaliplatin, are a class of metallodrugs widely used for the treatment of various malignancies including sarcomas and many solid epithelial cancers^1^. Given the widespread clinical use of these drugs, evaluation of the intake, distribution, and metabolism of these pharmaceutics within tumors and tumor models is important to study the potential shortcomings. Some deficiencies include inadequate penetration and non-specific binding.

In previous studies, imaging of Pt-based anti-cancer agents in biological samples has been visualized using a number of different techniques. Fluorescence imaging is frequently used and is highly sensitive, but requires the tagging of Pt complexes to fluorophores^2^. The addition of fluorophores can affect the distribution of the Pt species due to interactions of the conjugates with biological macromolecules^3^. Potential ligand exchange can also lead to loss of the fluorescent tag, resulting in incorrect distribution results. For these reasons, complementary imaging techniques are needed.

Additional analytical methods, including X-ray based techniques^4^,^5^, laser ablation inductively coupled plasma mass spectrometry (LA-ICP-MS)^6^,^7^,^8^, nano secondary ion mass spectrometry (nanoSIMS)^9^, and matrix-assisted laser desorption/ionization mass spectrometry (MALDI-MS)^10^, have also been employed to image the distribution of metallodrugs in biological tissues. Among these approaches, LA-ICP-MS has been the most widely utilized. LA-ICP-MS offers sufficiently high sensitivity in low μg/g range and improved spatial resolution at the submicrometer level. Another key advantage is that ICP-MS allows elemental visualization independent of the chemical binding partner and has been used to map the spatial distributions of metals in histologically heterogeneous structures^11^,^12^. However, this technique is limited to heteroatom-containing molecules only. As a result, another significant disadvantage is that ICP-MS fails to distinguish between the parent drug molecules and their metabolized species.

MALDI imaging mass spectrometry (IMS) is a popular alternative to ICP-MS. MALDI IMS analysis visualizes multiple mass to charge (*m/z)* ratios of molecules of interest simultaneously, and has been widely used for proteins, peptides, lipids, drugs and metabolites^13^. In a previous study, MALDI IMS was used to map the distribution of oxaliplatin and its metabolites in rat kidneys^10^. However, in other attempts, MALDI IMS has been found to suffer poor ionization and signal suppression by the matrix and the complexity of the biological samples, leading to false negatives. For example, LA-ICP-MS and MALDI IMS were combined to analyze patient tumor samples from colorectal or ovarian peritoneal carcinomatosis treated with Pt-based drugs^7^. In this study, cisplatin was only mapped by ICP-MS, as neither it nor any of its metabolites were detected in any of the MALDI IMS analyses.

In our past research efforts, we have also observed limited detection of Pt-based drugs by MALDI IMS. Optimization of sample preparation including change of matrix, matrix solvent composition, and matrix application methods did not help improve the sensitivity of the analysis. To overcome this challenge, we applied a derivatization reagent, diethyldithiocarbamate (DDTC), prior to matrix deposition to increase ionization efficiency of Pt-containing species. DDTC, a nucleophile sulfur-containing compound, is a chelating agent that forms metallic complexes with a number of metallic ions, such as copper, iron(II), iron(III), nickel, zinc, cadmium, Pt(II), and Pt(IV). In former studies, complexation with DDTC has been used in the quantification of Pt-based drugs to increase sensitivity and stability using liquid chromatography coupled to tandem mass spectrometry (LC-MS/MS)^14^,^15^,^16^,^17^. However, the application of DDTC has not been explored for direct on-tissue derivatization followed by MALDI imaging analysis. DDTC and Pt-DDTC conjugates have been shown to absorb UV light at 355 nm more efficiently than metallodrugs alone. Also, both DDTC and Pt-DDTC have been shown to co-crystallize well with MALDI matrices in organic solvents^18^. Therefore, we hypothesized that Pt-DDTC complexes would be more readily ionized for sensitive detection of Pt-containing drugs and their metabolites on tissue slices.

In this present work, we evaluated the distribution of oxaliplatin in hyperthermic intraperitoneal chemotherapy (HIPEC)-like treated three-dimensional (3D) multicellular tumor spheroids (MCTS). HIPEC is a treatment approach involving a local application of high doses of heated chemotherapy drugs^19^,^20^. It is performed after cytoreductive surgery to remove tumors from the abdominal cavity^19^. HIPEC allows application of a more intense dose of chemotherapy, with less systemic toxicity for patients as the drugs stay within the abdominal area^21^. In HIPEC, the drugs are heated to improve both efficacy and penetration, making them more effective in killing cancer cells^22^. Better monitoring of the penetration, distribution, metabolism, and clearance of HIPEC-administered therapeutics would be enormously beneficial in understanding the tumor response and optimization of this increasingly popular therapeutic approach.

In this study, 3D MCTS were used as the *in vitro* tumor model. MCTS are an attractive and valuable preclinical system for the screening of pharmaceutical compounds^23^,^24^. They recapitulate many features of solid tumors *in vivo*, such as the development of 3D cellular organization (e.g. outer proliferating and intermediate quiescent cell zones, hypoxic and necrotic core regions), tumor microenvironment, 3D cell-cell interactions, as well as the accumulation of an extracellular matrix that may affect drug behavior. In a previous study, we successfully mapped the distribution of irinotecan and its metabolites in this model system using MALDI IMS complemented with LC-MS/MS quantification^25^. In the current study, we extend our MALDI IMS based analyses to map the distribution of Pt-based drugs, and in particular, the therapeutics oxaliplatin, carboplatin, and cisplatin. We are developing a simple, rapid, and sensitive approach to study the drug response and localization of Pt-based therapeutics within MCTS using DDTC derivatization, followed by MALDI IMS or LC-MS/MS analysis, to better understand and optimize HIPEC treatment. A schematic illustration for this approach is shown in Fig. 1.

## Experimental

Detailed discussion can be found in SI materials and methods.

### MCTS Culture and HIPEC-like Treatment with Oxaliplatin

MCTS were generated using the colon carcinoma cell line HCT 116 cells (ATCC, Manassas, VA) as previously described^25^,^26^. MCTS on day 13 were incubated in pre-warmed oxaliplatin solution (12.6 mM, 42 °C) or in nanoPure water for 15 min, 30 min or 90 min. At the end of the incubation of 30 min or 90 min, some of the MCTS were transferred to a new agarose-coated 96-well plate, and cultured in drug-free medium for different lengths of time (2, 6, 12, and 24 h) to further study the post treatment effects and drug elimination. Untreated MCTS were cultured in parallel as references.

### On-tissue Derivatization and Sample Preparation for MALDI IMS Analysis

After specific incubation times, the MCTS were then harvested and sectioned into 12 μm slices^25^. 1% DDTC was prepared in 0.1 M NaOH solution and applied onto the sample as a very fine mist using an airbrush. The sprayed slide was placed in a Petri dish containing a moist Kim-Wipe and the plate was incubated at 40 °C for 5 min. This whole process was repeated six times. Matrix (*alpha*-cyano-4-hydroxycinnamic acid, CHCA) was then applied onto the sample.

### MALDI IMS and Data Analysis

Mass spectra were acquired using an UltrafleXtreme TOF/TOF mass spectrometer (Bruker Daltonik, Bremen, Germany). The ion images were visualized using FlexImaging (ver. 4.0; Bruker Daltonics) or analyzed with SCiLS Lab (ver. 2015b; Bremen, Germany).

### Sample Preparation for UPLC-MS/MS Analysis

HIPEC-like treated and untreated control MCTS were prepared as described above. Cells from the outer, intermediate, and core regions of these MCTS were fractionated using serial trypsinization^27^. Small molecules were then extracted and nickel chloride (Sigma, St. Louis, MO) was added as the internal standard. The mixture was then derivatized with DDTC, and extracted with ethyl acetate/ *n*-hexane (EA/Hex, 1:1, v/v)^17^. To analyze drug efflux, cell culture medium was collected at different time points and small molecules were then extracted and derivatized using the same sample pretreatment procedure. All treatment conditions were performed with four replicates. For quantification of total Pt concentration, fractionated cells, spiked with the internal standard, were wet-ashed with concentrated nitric acid (HNO_3_)^18^, followed by DDTC derivatization and EA/Hex extraction.

### Chromatographic and Mass Spectrometric Conditions for Multiple Reaction Monitoring (MRM)

A Waters Acquity UPLC system (Milford, MA) was used. All MRM mass spectrometric experiments were performed with a Waters TQD tandem quadrupole detector (Milford, MA) monitored with MassLynx MS software. Samples were analyzed in positive ESI mode with MRM of the transitions *m/z* 492 → *m/z* 116 for Pt(DDTC)_2_, *m/z* 640 → *m/z* 492 for Pt(DDTC)_3_, and *m/z* 355 → *m/z* 116 for the internal standard nickel complex Ni(DDTC)_2_.

## Results and Discussion

### Reactions of oxaliplatin

Oxaliplatin, or [Pt(R,R-DACH)(oxalate)] (R,R-DACH = R,R-cyclohexane-1,2-diamine), is frequently administrated in the treatment of colorectal cancer^28^. Oxaliplatin can be non-enzymatically converted by reaction with water and chloride to [Pt(DACH)Cl_2_], [Pt(DACH)Cl(OH)], and [Pt(DACH)(OH)_2_]^29^. Pt(ΙΙ) drugs are known to form adducts with nucleobases resulting in DNA cross-links^30^, which inhibits DNA synthesis and repair, which eventually leads to apoptosis, or programmed cell death. Pt compounds also have a high affinity towards sulfur-containing molecules. The biotransformation of oxaliplatin from reactions with cysteine, glutathione, and methionine at physiological pH has shown that oxaliplatin complexes could be formed via direct displacement of the labile leaving ligand without prior aquation^31^,^32^. Previous studies demonstrated that Pt-methionine complexes may slowly rearrange to Pt-guanosine 5- monophosphate complexes due to trans-labilization and more favorable thermodynamics^33^,^34^. This rearrangement does not seem to happen for oxaliplatin because of the more stable DACH ligand^35^. Therefore, intact oxaliplatin is the main cytotoxic component, whereas the sulfur-Pt complexes are most likely inactive and non-cytotoxic products.

### Detection of oxaliplatin metabolites in MCTS without DDTC derivatization

First, the cell viabilities of oxaliplatin-treated MCTS were analyzed as indicators of drug activity (Supplemental Figure 1). The percentage of viable cells decreased to 64.0 ± 6.5% or to 49.1 ± 6.0% in MCTS 48 h after a 30 min or a 90 min HIPEC-like treatment, respectively. This result was consistent using different viability assay methods. This difference in average viability suggests an increased cellular uptake of oxaliplatin in the 90 min treated MCTS compared to those treated for 30 min.

MALDI IMS was then performed to map oxaliplatin and its metabolites in MCTS. The Pt-containing compounds were assessed at the *m/z* values corresponding to the major isotopes of Pt, ^194^Pt, ^195^Pt, ^196^Pt and ^198^Pt, with relative abundances of 32.9, 33.9, 25.9 and 7.2%, respectively^7^. Comparison of spectra acquired from oxaliplatin treated and control MCTS revealed an isotope cluster at *m/z* 456–460 in the 90 min dosed samples. This signal cluster corresponded to the predicted masses of the monomethionine-Pt(DACH) complex, which is one of the metabolites of oxaliplatin. The presence of this metabolite indicates deactivation of the drug. As shown in Supplemental Figure 2, the oxaliplatin-methionine metabolite is mainly localized in the core region of MCTS. This accumulation may be related to the up-regulation of thiols by cells in response to high hypoxic and oxidative stress^36^. Alternatively, thiol-adducts are known to be exported out of cells through detoxification mechanisms^37^. Methionine may accumulate with other ions and waste products, such as lactate in the necrotic region of MCTS, with ample extracellular space and possibly a slow draining mechanism.

While the Pt(DACH)(Met) complex was detected without DDTC derivatization, no other drug-related species including the intact oxaliplatin molecule, were detected by MALDI in either positive or negative ion mode. This lack of detectable signals may be due to insufficient ionization of Pt-containing compounds. Also, the Pt(DACH)(Met) complex was only detected in the 90 min treated MCTS and not in the 30 min specimens or any samples at other post-treatment time points. Enhancement of Pt-species ionization efficiency and improvement of detection sensitivity were needed to reveal the actual penetration of oxaliplatin and the localization of its metabolites.

### Distribution of Pt-related species in oxaliplatin treated MCTS with DDTC derivatization

**Figure 2:**
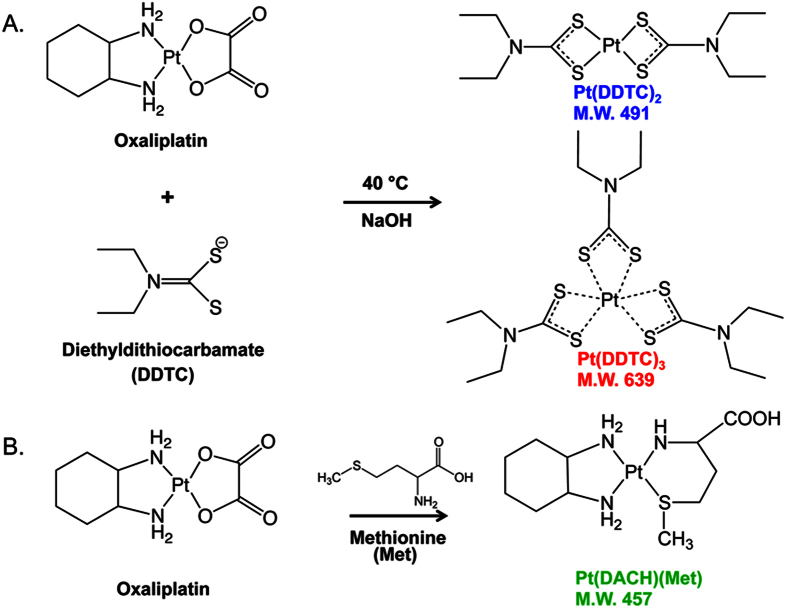
Derivatization of platinum drugs with N,N-diethyldithiocarbamate (DDTC). (**A**) DDTC abstracts Pt under basic, heated reaction conditions to form either a dimer, Pt(DDTC)2 (*m/z* 491), or a trimer compound, Pt(DDTC)3 (*m/z* 639). (**B**) Oxaliplatin reacts with methionine to form Pt(DACH)(Met), (*m/z* 457).

To increase ionization of Pt species, DDTC derivatization was used. In the derivatization, Pt is complexed with DDTC, which abstracts the metal and forms either a dimer or trimer complex (Fig. 2). In the experimental approach, DDTC is incubated with the Pt compound in a basic solution to allow chelation of the metal. DDTC can complex with the Pt as a dimer or trimer cluster (Fig. 2A). The dimer cluster (two DDTC chelated to one Pt) has a mass of 491 Da while the trimer cluster (three DDTC molecules chelated to one Pt) has a mass of 639 Da. Another common reaction is the formation of the Pt(DACH)(Met) metabolite, formed when the amino acid methionine reacts with the Pt compound (Fig. 2B). As described in the last section, unlike the intact drugs, the monomethionine metabolite is readily detectable by MALDI analysis. The monomethionine compound of oxaliplatin, known as Pt(DACH)(Met), is shown in Fig. 2B and has a mass of 457 Da.

**Figure 3:**
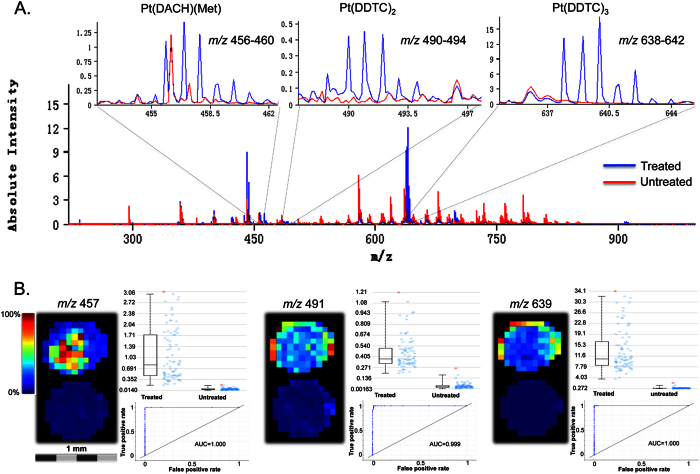
MALDI IMS detection of oxaliplatin related species in MCTS through DDTC derivatization. (**A**) Overlapped spectral profiles for oxaliplatin-treated (blue) and untreated (red) MCTS. The isotope clusters at *m/z* 456 to 460, 490 to 494, and 638 to 642 were assigned to the metabolite monomethionine-Pt(DACH), Pt(DDTC)2, and Pt(DDTC)3 complexes, respectively. (**B**) Ion images, box and ROC plots reveal selected signals (*m/z* = 457, 491, and 639) specifically distinguishing oxaliplatin-treated MCTS from untreated MCTS. Please note that the Pt drugs are only detectable with DDTC.

DDTC was applied on MCTS slices to convert free Pt-containing molecules to hydrophobic chelate complexes, followed by the spraying of CHCA matrix. After MALDI IMS analysis, spectra and images acquired from the oxaliplatin treated and untreated MCTS were analyzed by calculating co-localized and anti-correlated *m/z* values as well as using the ROC tool to find discriminating *m/z* signals. ROC analysis is a univariate measurement determining how well a selected *m/z* value distinguishes two different samples^38^. It includes estimating specificity and sensitivity values for a trivial threshold classifier, followed by plotting a curve for the computed results. The area under the ROC curve (AUC value) represents the discrimination power of the *m/z* signal. A perfect discrimination would yield an AUC equal to 1 (abundant in group 1) or 0 (abundant in group 2). The closer the AUC to 0.5, the less suitable the *m/z* value is to be used as a univariate criterion. In the analysis, several clusters of signals corresponding to isotopic distribution of Pt were found to be highly discriminating between the 90 min oxaliplatin treated and untreated MCTS (AUC value > 0.95). Among these values, *m/z* 456–460, 490–494, and 638–642 were assigned to Pt(DACH)(Met), Pt(DDTC)_2_, Pt(DDTC)_3_ respectively (Fig. 3A), while *m/z* 512–516 and 519–523 need further analysis to characterize their structures. The ion images, relative intensity plots, and ROC curves of *m/z* 457 [Pt(DACH)(Met)]^+^, *m/z* 491 [Pt(DDTC)_2_]^+^, and *m/z* 639 [Pt(DDTC)_3_]^+^ in Fig. 3B show the detection of these molecules only in oxaliplatin treated, but not in the untreated MCTS.

The chelation of oxaliplatin with the sulfur nucleophile DDTC was efficient and rapid due to strong Pt-S bond formation. The resulting chelated complexes [Pt(DDTC)_2_]^+^ and [Pt(DDTC)_3_]^+^ were readily ionized and could be detected by MALDI MS with high sensitivity, even in the absence of CHCA matrix (Supplemental Figure 3A). However, the signals were less than 1% of those with the application of a suitable amount of CHCA matrix. Therefore, matrices were still needed to guarantee a sensitive detection of Pt-DDTC compounds. Moreover, derivatization with DDTC resulted in an increase of the signals from [Pt(DACH)(Met)]^+^ (Supplemental Figure 3B). It may be that DDTC allowed more efficient absorption of laser light, leading to a more effective energy transfer to the analyte Pt(DACH)(Met). In short, this derivatization was necessary for sensitive detection of the Pt-containing drug molecules.

To further evaluate the different spatial localization of these Pt-related molecules, pLSA component analysis was used to analyze the datasets. Three, five and seven pLSA components were tested in advance, and an optimal number of five was used to properly represent the distribution pattern within the sample. In a 90-min treated MCTS, the loading plots and score structures of the first two components are readily distinguishable (Supplemental Figure 4A). The results of the pLSA analysis indicate the distinct spatial sample components and their corresponding mass distributions in the MCTS. Signals matching the *m/z* values of Pt(DDTC)_2_ and Pt(DDTC)_3_ were amongst the highest loading scores in component 1, which described a distribution pattern of molecules present mainly in the outer region of MCTS. In contrast, the *m/z* values from Pt(DACH)(Met) contributed predominately to component 2, which illustrated a principal localization of species in the central area of the MCTS. Correlation plots of *m/z* values in Supplemental Figure 4B confirmed the co-localization of [Pt(DDTC)_2_]^+^ (*m/z* 491) and [Pt(DDTC)_3_]^+^(*m/z* 639) with a correlation value of 0.715 compared to a threshold of 0.5 (p ≤ 0.05). By comparison, the correlation of [Pt(DACH)(Met)]^+^ (*m/z* 457) and [Pt(DDTC)_3_]^+^(*m/z* 639) was insignificant with a correlation of 0.00139. Finally, ion images reconstructed from MALDI IMS results demonstrated the same distribution pattern found in the above statistical analysis (Supplemental Figure 4C), indicating that the [Pt(DACH)(Met)]^+^ has distinct localization compared with [Pt(DDTC)_2_]^+^ and [Pt(DDTC)_3_]^+^.

It has previously been reported that Pt drugs were observed to accumulate in the hypoxic core region as well as the periphery area of MCTS using LA-ICP-MS after 96 h treatment (*6*). However, this methodology failed to distinguish the parent drug from its metabolites. Our results with MALDI IMS suggest that with the 90-min HIPEC-like treatment of oxaliplatin, most of the free drug is present in the outer region of the MCTS. While some drug molecules did reach the core area, they rapidly reacted with methionine, and accumulated as an inactive metabolite. Derivatization with DDTC enabled sensitive detection of free drug molecules and did not reverse the binding of oxaliplatin-methionine, thus allowing the mapping of this metabolite at the same time. Pt-molecules were also successfully detected through DDTC derivatization in cisplatin or carboplatin treated MCTS as shown in Supplemental Figure 5. Both of the drugs were found to reach and accumulate in the core region of MCTS with a 48 h treatment.

A copper-DDTC complex, Cu(DDTC)_2_. formed following DDTC derivatization was also detected by MALDI MS (Supplemental Fig. 6A). It is known that cancer cells concentrate high levels of copper, which is important for both angiogenesis and metastasis^39^. HCT 116 cells have been shown to accumulate 7-fold higher levels of copper compared to normal cells^39^. Our MALDI IMS results show that Cu(DDTC)_2_ was slightly more concentrated in the core region of the MCTS. This fact is consistent with a previous study showing elevated Cu concentration in the necrotic core of MCTS using X-ray fluorescence microscopy mapping^36^. Cu levels may be elevated in the hypoxic core region of MCTS in response to oxidative stress to protect cells from damage. Alternatively, hypoxia was found to upregulate the ATP7A Cu-efflux transporter to reduce free Cu catalyzing the formation of reactive oxygen species^40^. The elevated Cu concentration in the central region may be due to cells exporting intracellular Cu into the extracellular matrix, resulting in its accumulation in the core of the MCTS, along with other waste metabolic products.

### Time Course of Oxaliplatin Diffusion and Clearance in MCTS

**Figure 4:**
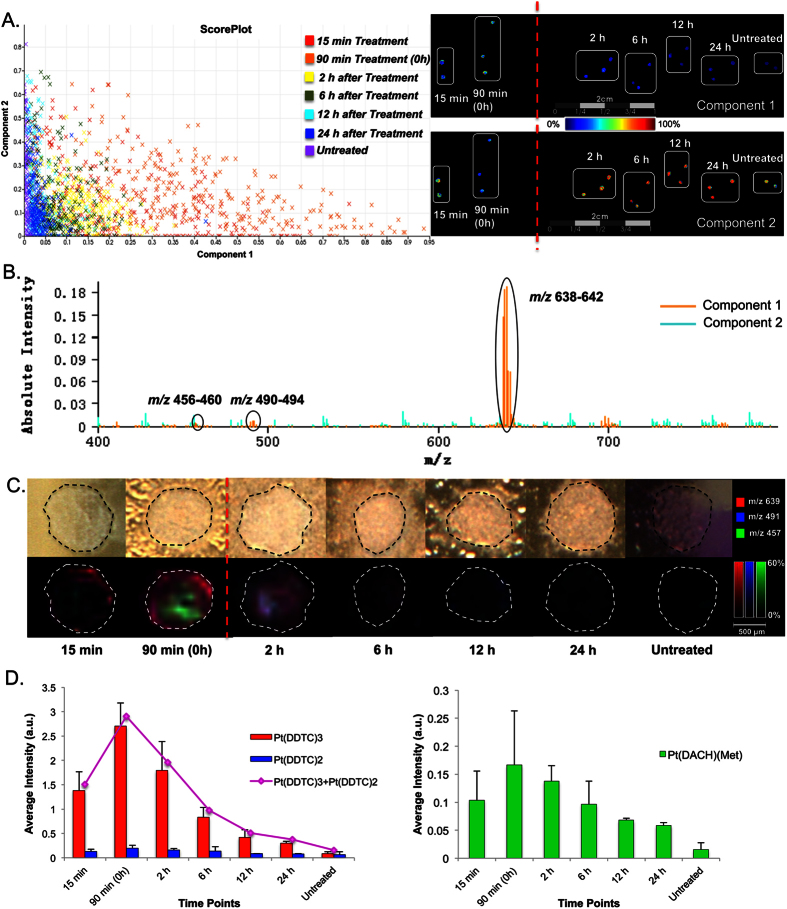
HIPEC treatment of colorectal cancer MCTS with oxaliplatin. (**A**) Probabilistic Latent Semantic Analysis (pLSA) of MCTS (with DDTC derivatization). The MCTS were treated for 15 or 90 min. Drug clearance was evaluated at 2, 6, 12, and 24 h post 90 min treatment. pLSA was able to separate spectra from oxaliplatin treated and untreated MCTS in the first component as shown in the Score Plot. The score images show that Component 1 and Component 2 depict completely different distribution patterns in the dataset. (**B**) Loading plots illustrate the Pt(DDTC)2, Pt(DDTC)3 signals (*m/z* 491 and 639) and the Pt(DACH)(Met) metabolite, also known as the monomethionine metabolite (*m/z* 457), are in the first component. (**C**) MALDI IMS images of HIPEC treated MCTS and the relative distribution of Pt species. (**D**) Average signal intensities of Pt species from MALDI IMS results.

Drug penetration was analyzed with different HIPEC-like treatment times of 15 min, 30 min, and 90 min. Oxaliplatin and its metabolite redistribution during washing was also studied by transferring MCTS to drug-free media and harvesting at different time points following the treatment. pLSA was used to compare spectra acquired by MALDI IMS for the examination of trends in the dataset (Fig. 4A and B). Visualization of Component 1 identified a pattern with the highest distribution in 90 min treated samples, and steadily decreased spectral similarities in MCTS following the treatment. Loading plots showed that this spectral feature within the dataset was largely associated with drug-related molecules Pt(DACH)(Met), Pt(DDTC)_2_, Pt(DDTC)_3_. Figure 4C depicts the co-localized ion images of *m/z* 457 [Pt(DACH)(Met)]^+^, *m/z* 491 [Pt(DDTC)_2_]^+^, and *m/z* 639 [Pt(DDTC)_3_]^+^ at different time points. It was found that even at the earliest time point, 15 min, the oxaliplatin-related molecules were detectable and observed to localize at the outer region of MCTS, indicating limited penetration at this time point. Continuous drug exposure for 90 min resulted in an enhancement of drug penetration and increased formation of Pt(DACH)(Met) metabolite. After 2 hours of washing, oxaliplatin-related species including the metabolite, showed a fast rate of removal from MCTS resulting in a negligible drug signal detected after 24 hours.

Average peak intensities of these molecules at different time points were calculated and compared in Fig. 4D. Pt concentration peaked at 90 min, and dropped very rapidly. After 24 hours of washing, the signals from Pt(DDTC)_3_ and Pt(DACH)(Met) were reduced to approximately 11 and 34% of peak levels, respectively. Peak intensity from Cu(DDTC)_2_ was used as an internal reference showing no significant difference between samples (Supplemental Figure 6B). Similar trends of diffusion and clearance of oxaliplatin were observed in 30 min treated MCTS (Supplemental Figure 7).

### Quantification of Free Oxaliplatin-related Molecules in Different Regions of MCTS by UPLC-MRM

At the preliminary stage of the method development, quantification of oxaliplatin without derivatization was tested. However, the unchanged oxaliplatin presented very poor retention on either reverse phase columns or hydrophilic interaction chromatography columns. In addition, the signal response of the MRM transition for oxaliplatin was below the threshold to fulfill quantification requirements. Therefore, DDTC derivatization was performed to increase selectivity, sensitivity, and stability for measurement of Pt drug.

#### Linearity, Accuracy and Precision

Calibration curves were constructed for Pt(DDTC)_2_ and Pt(DDTC)_3_ in MCTS extracts, wet-ashed MCTS or medium. The calculated peak area ratios of calibration standards to Ni(DDTC)_2_ were proportional to the concentration of the analytes over selected concentration range, 50.3–6292.6 nM (Supplemental Figure 8). The linearity of the assay was determined by generation of a regression line using least square analysis. As evident from the coefficients of ≥0.99 for all runs, the calibration curves met acceptance criteria at the tested concentrations. Accuracy and precision results for the calibration samples are provided in Supplemental Table 1.

QC samples were prepared in five replicates at four different concentrations by independently weighing separate amounts of the standards used to construct calibration curves. To avoid interference, double blank (blank matrix only) and QC (blank matrix spiked with internal standard only) were also prepared and analyzed. The intra-/inter-batch accuracy was from 94.0/97.2% to 107.2/107.2%, with the precision of R.S.D. ≤ 16.5/11.0% in MCTS extracts, and from 95.3/118.9% to 97.2/117.4%, with the precision of R.S.D. ≤ 18.5/16.8% in culture medium, and from 89.3/91.2% to 106.0/104.9%, with precision of R.S.D. ≤ 17.4/16.6% in wet-ashed MCTS. Both precision (R.S.D. ≤ 15%) and accuracy (85–115%) were acceptable for low, medium and high concentration QC samples, with satisfactory precision (R.S.D. ≤ 20%) and accuracy (80–120%) for lower limit of quantification QC samples. The accuracy and precision data is shown in Supplemental Table 2.

#### Matrix Effect and Stability

The matrix effect was investigated by comparing the peak areas of samples prepared in MCTS extracts, medium or wet-ashed MCTS with the peak areas of neat solutions at the same concentrations. A small change in signal intensity was observed for Pt-DDTC in MCTS extracts and culture medium, but by using Ni-DDTC as the internal standard, the observed matrix effect was corrected. The signal changed less than 10% for standards prepared in MCTS extracts or in culture medium (Supplemental Figure 9), indicating that the matrix effect did not significantly affect the signal response of the target analyte Pt-DDTC. Wet-ashed MCTS sample presented a relatively higher ion enhancement with an average matrix effect of 136.9%, but the use of an internal standard helped compensate for this bias.

Samples either in MCTS extracts or medium stored at −20 °C were tested over a 14 day period. Pt-DDTC showed no sign of degradation. QC samples in both matrices were allowed to stand at room temperature for at least 24 h and maintained the nominal starting concentration. This is much more reliable compared to the quantification of oxaliplatin without using derivatization, in which the degradation of the drug in injection solution and mobile phases was a critical issue to examine.

#### Analysis of Free Oxaliplatin-related Molecules and Total Pt Concentration in MCTS and Culture Medium

**Figure 5:**
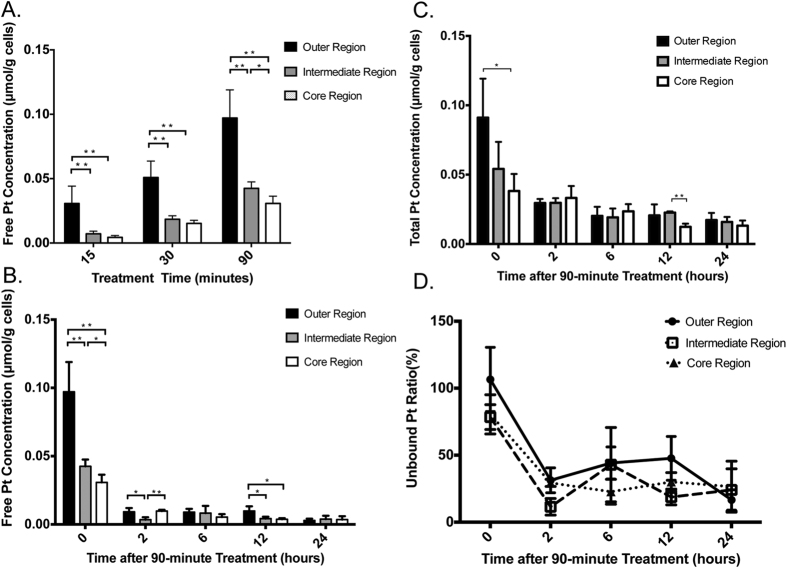
Quantification of free Pt-containing species or total Pt concentration in different regions of HIPEC treated MCTS. (**A**) Drug penetration in 15-min, 30-min or 90-min treated MCTS. (**B**) Free drug clearance for 90-min treated MCTS. (**C**) Total drug clearance for 90-min treated MCTS. (**D**) Unbound Pt ratio following 90-min treated MCTS. *p < 0.05, **p < 0.01.

The validated method was used to investigate the distribution of free oxaliplatin molecules in MCTS. As shown in Fig 5, the total amount of oxaliplatin steadily increased in the MCTS with the drug incubation time. With 90 min of incubation, Pt-containing molecules mainly accumulated in the outer and intermediate regions, which was consistent with our previous MALDI IMS results. During drug clearance, the concentration of free oxaliplatin-related species decreased rapidly in just 2 hours. After 24 hours, only 5.4% (Supplemental Figure 10A) and 7.0% of the initial levels were present in the 30 min and 90 min treated spheroids, respectively. This change may be due to a rapid efflux of the free drug molecules from the MCTS, resulting in reduced concentration in the MCTS. Binding to endogenous amino acids, proteins and DNA is another factor that influences the free drug amount in cells. Quantification of total Pt concentration shows that 2 hours after the 90 min treatment about 48.3% Pt molecules was cleared from cells. A further decrease in the Pt concentration to 35.8% of the original was observed at the 6 hour time point (Supplemental Figure 10B). After 24 hours, there was still 30.5% Pt remaining in cells. The unbound (free) Pt ratio decreased from 83.0 ± 10.2% to 19.3 ± 2.2% in 24 hours (Supplemental Figure 11), indicating that most of the Pt drug in MCTS was unbound immediately after the HIPEC-like treatment. However, for the Pt remaining in cells after washing, about 80% was in a bound state.

Measurement of the Pt level was also performed in culture medium at different time points (Supplemental Figure 10C). Surprisingly, concentration of free drug molecules increased drastically in the first 2 h, followed by a steady decrease afterwards. We hypothesized that the released free drug species may penetrate into the agarose gel, causing the decreased of drug concentration in the media. It was also possible that some Pt-containing molecules diffused back into the MCTS and were metabolized.

## Conclusions

In this manuscript, we demonstrate on-tissue derivatization of Pt to enable MALDI IMS ionization and sensitive analysis of Pt-containing drug species. We demonstrate the detection of oxaliplatin, cisplatin, and carboplatin drugs in colorectal MCTS. In combination with a UPLC-MRM method, this methodology was quantitative and can be easily implemented for the spatial mapping of Pt-drugs, providing critical information about their permeability and metabolic properties. This approach has great potential to be applied in testing of other metallodrugs or endogenous metal ions that can chelate with DDTC. The resulting changes of other biological macromolecules following the drug treatment could also be studied.

## Additional Information

**How to cite this article**: Liu, X. and Hummon, A. B. Chemical Imaging of Platinum-Based Drugs and their Metabolites. *Sci. Rep.* **6**, 38507; doi: 10.1038/srep38507 (2016).

**Publisher's note:** Springer Nature remains neutral with regard to jurisdictional claims in published maps and institutional affiliations.

## Supplementary Material

Supplementary Information

## Figures and Tables

**Figure 1:**
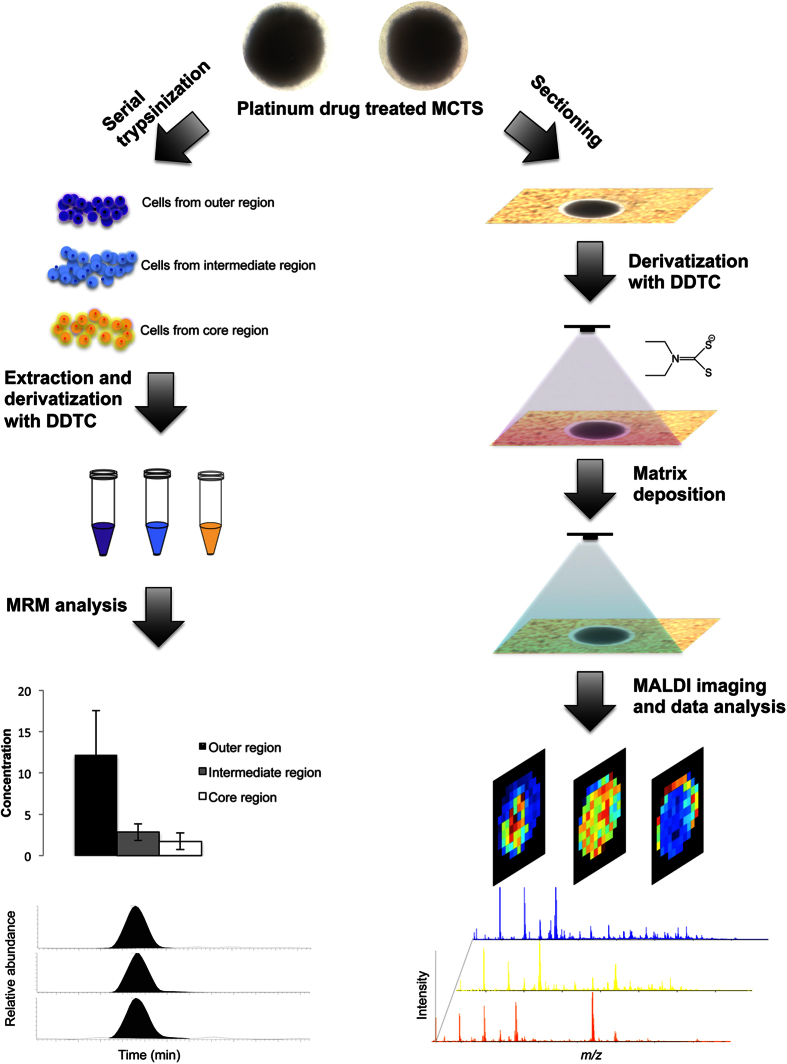
Overall workflow for spatial analysis by IMS and quantitative analysis by LC-MS/MS.
