# Supplementary material for: Chemical Imaging of Platinum-Based Drugs and their Metabolites
Source: Sci Rep. 2016 Dec 5;6:38507. doi: 10.1038/srep38507 (PMC5137023; doi:10.1038/srep38507)
Supplement: Supplementary Information [file srep38507-s1.doc]

**Supplemental Information (SI)**

**Chemical Imaging of Platinum-Based Drugs and their Metabolites**

Xin Liu^1^, and Amanda B. Hummon ^1^*

^1^Department of Chemistry and Biochemistry

University of Notre Dame

McCourtney Hall

Notre Dame, IN 46556, USA

*Corresponding author

ahummon@nd.edu

Phone: 574-631-0583

Fax: 574-631-6652

**This file contains:**

- Complete Materials and Methods
- Supplemental Figure 1: Microscopic images and viability of MCTS following HIPEC-like oxaliplatin treatment.
- Supplemental Figure 2: MALDI IMS results of 90 min treated and untreated MCTS without DDTC derivatization.
- Supplemental Figure 3: Increased signal intensities of Pt-related species using DDTC derivatization and application of matrix.
- Supplemental Figure 4: Statistical analysis to evaluate different localization of Pt species in 90-min HIPEC treated MCTS.
- Supplemental Figure 5: MALDI IMS results with DDTC derivatization of cisplatin or carboplatin treated spheroids.
- Supplemental Figure 6: Detection of Cu(DDTC)_2_ by MALDI IMS.
- Supplemental Figure 7: MALDI IMS results of 30 min HIPEC treated MCTS.
- Supplemental Figure 8: Calibration curve and reproducibility of internal standard.
- Supplemental Table 1: Precision and accuracy of the assay for oxaliplatin with the calibration samples.
- Supplemental Table 2: Precision and accuracy of the assay for oxaliplatin with the quality control samples.
- Supplemental Figure 9: Matrix effect
- Supplemental Figure 10: Quantification of free Pt or total Pt molecules in MCTS.
- Supplemental Figure 11: Unbound Pt ratio in MCTS.

**MATERIALS AND METHODS**

*Cell Line and MCTS Culture*

The colon carcinoma cell line HCT 116 (ATCC, Manassas, VA) was grown in McCoy’s 5A cell culture medium (Life Technologies, Grand Island, NY) supplemented with 10% fetal bovine serum (Thermo Scientific, Gaithersburg, MD). Cells were maintained in 5% CO_2_ at 37 °C and passed every five days. Cell lines were used within three months after resuscitation of frozen aliquots thawed from liquid nitrogen. The provider assured the authentication of these cell lines.

MCTS were generated by loading suspensions of HCT 116 cells at a density of 7000 cells in 200 μL medium into each well of agarose-coated 96-well plate. After an initiation interval of four days, fifty percent of the culture volume was replaced with fresh medium every two days thereafter. A MCTS diameter of about 1 mm was reached after twelve days in culture.

*HIPEC-like Treatment with Oxaliplatin in MCTS*

Oxaliplatin was purchased from Sigma (St. Louis, MO) or Santa Cruz Biotechnology (Santa Cruz, CA). MCTS on day 13 were incubated in pre-warmed oxaliplatin solution (5 mg/mL, 42 °C) or in nanoPure water for 15 min, 30 min or 90 min, which was designed to mimic HIPEC treatment conditions in patients diagnosed with peritoneal carcinomatosis from colorectal cancer. At the end of the incubation of 30 min or 90 min, some of the MCTS were transferred to a new agarose-coated 96-well plate, and cultured in drug-free medium for different lengths of time (2, 6, 12, and 24 h) to further study the post treatment effects and drug elimination in MCTS. Untreated MCTS were cultured in parallel as references. After 48 h, cell viability and metabolic activity were measured using the Cell Titer-Blue Viability assay as previously described. Acid phosphates assay kit (Abcam, Cambridge, UK) was also used as manufacturer’s instructions to confirm the viability result.

*On-tissue Derivatization and Sample Preparation for MALDI IMS Analysis*

After specific incubation times, the medium was aspirated and the MCTS were washed with 1X phosphate buffered saline (PBS) twice respectively. The MCTS were then harvested and sectioned into 12 μm slices using the gelatin assisted sectioning method, and thaw-mounted on the same slide to avoid sample preparation variance. 1% DDTC was prepared in 0.1 M NaOH solution and applied onto the sample as a very fine mist using an airbrush. The sprayed slide was placed in a Petri dish containing a moist Kim-Wipe and the plate was incubated at 40 °C for 5 min. This whole process was repeated six times for the complete chelation reaction. The slides were allowed to cool and dry in a vacuum desiccator for 30 min. Matrix (*alpha*-cyano-4-hydroxycinnamic acid, CHCA) was prepared in 50:50 HPLC-grade acetonitrile (ACN) -water with 0.1% trifluoroacetic acid (TFA) (EMD, Billerica, MA) to yield a final concentration of 10 mg/mL, and was then sprayed onto the sample. The application was monitored under an optical microscope with 10× magnification.

### *MALDI IMS and Data Analysis*

### Mass spectra were acquired using an UltrafleXtreme TOF/TOF mass spectrometer (Bruker Daltonik, Bremen, Germany) equipped with smartbeam II Nd:YAG 355 nm laser operating in reflectron, positive mode at 1000 Hz, or an AutoFlex III smartbeam TOF/TOF mass spectrometer operating at 100 Hz. For IMS data acquisition, 800 laser shots were accumulated per array position with a lateral resolution of 75 μm diameter using the “large” focus setting under optimized delayed extraction conditions, in the mass range of *m/z* 200-1000 Da. External calibration was performed using a custom peptide mixture by spotting the standards on a region without gelatin near the MCTS section.

### The ion images were visualized using FlexImaging (ver. 4.0; Bruker Daltonics) or analyzed with SCiLS Lab (ver. 2015b; Bremen, Germany). Raw data was imported into SCiLS Lab software and processed as follows: baseline removal with TopHat algorithm, normalization with total ion current (TIC) method, peak picking using orthogonal matching pursuit algorithm, peak alignment to correct for slight mass-to-charge variation. For supervised data analysis, peaks that discriminated drug treated and untreated MCTS were elucidated by means of receiver operating characteristic (ROC) curves or Pearson correlation analysis. For unsupervised statistical analysis, probabilistic latent semantic analysis (pLSA) was used to assess the overall features in the sample. In comparison to principal component analysis (PCA), a particular advantage of pLSA is that the loadings are nonnegative, and can be interpreted as mass spectra intensities. The results of the pLSA therefore represent real sample components and their respective spatial localization. Different numbers of pLSA components were always tested in advance by running three, five, and seven components to determine the optimal number to evaluate distribution patterns within the sample. In all analyses, the interval width was set to 0.05% Da.

*Sample Preparation for UPLC-MS/MS Analysis*

HIPEC-like treated and untreated control MCTS were prepared as described above. Thirty MCTS were harvested for each time point and washed twice with PBS. Cells from the outer, intermediate, and core regions of these MCTS were fractionated using serial trypsinization. Cell pellets from each of the populations were weighed on a balance. Small molecules were then extracted by adding 500 μL acetonitrile (ACN) containing 100 ng internal standard (nickel chloride, 10 μL from 10 μg/mL solution), from Sigma (St. Louis, MO). The mixture was thoroughly vortexed, sonicated (the tube was refrigerated in an ice bath), and centrifuged at 13,500 rpm for 15 min at 4 °C. The supernatant was collected into another Eppendorf tube, and evaporated to dryness under vacuum in a SpeedVac concentrator, followed by reconstitution in 200 μL water. After addition of 80 μL 1% DDTC in 0.1 M NaOH, samples were placed in a 40 °C water bath. The mixture was incubated for 45 min, and then extracted with 500 μL of ethyl acetate/ *n*-hexane (EA/Hex, 1:1, v/v) by vortex mixing for 5 min (*17)*. The tube was then centrifuged at 12,000 rpm for 5 min at 4 °C. Thereafter, the supernatant was transferred to a new Eppendorf tube and evaporated to dryness at 40 °C. Following evaporation, the mixture was resuspended with 200 μL of ACN/H_2_O (80:20, v/v) and vortex-shaken for 5 min. Finally, 20 μL was injected into the LC-MS/MS system. To analyze drug efflux, 100 μL of cell culture medium was collected at different time points and small molecules were then extracted and derivatized using the same sample pretreatment procedure. All treatment conditions were performed with four replicates. For quantification of total Pt concentration, cells to which the internal standard had been added was mixed with 10 μL of concentrated HNO_3_ and was wet-ashed in 85 ^o^C for 8h. 5 M NaOH solution was added to adjusted the pH of the solution to 4-6. The volume was adjusted to 200 μL, and the solution was centrifuged at 10,000*g* for 10 min to remove precipitates. And then the supernatant was placed in a new tube, and 50 μL of 0.1 M DDTC solution was added. The mixture was incubated at 40 °C for 45 min, followed by the same extraction procedure described above.

*Calibration Curve and Method Validation*

Stock solutions (1 mg/mL) of oxaliplatin and nickel chloride were prepared in water. Calibration samples were prepared by spiking oxaliplatin and nickel chloride into drug-free control MCTS extracts or culture medium. For quantification of total Pt concentration, oxaliplatin with internal standard was spiked in control MCTS and wet-ashed. The following concentrations of oxaliplatin were prepared: 50.3, 125.9, 251.7, 503.4, 1258.5, 2517.1, 5034.1 (wet-ashed sample), or 6292.6 nM (MCTS extracts or culture medium), with the internal standard concentration of 3.86 μM. The standards were derivatized the same way described above. All solutions were stored at - 80 ^o^C under dark conditions to remain stable.

Separate quality control (QC) samples were prepared independent of those used for preparing calibration curves at concentrations of 50.3, 251.7, 1258.5, 6292.6 nM for oxaliplatin in MCTS lysates or in culture medium, and at concentrations of 50.3, 251.7, 1258.5, 5034.1 nM in wet-ashed MCTS. They were prepared using the same procedure as the drug treated samples. Intra-batch (n=4 or 5) and inter-batch (n=12 or 15) accuracy and precision were investigated.

*Chromatographic and Mass Spectrometric Conditions for Multiple Reaction Monitoring (MRM)*

A Waters Acquity UPLC system (Milford, MA) equipped with a binary solvent manager, an autosampler, and a photodiode array detector was used. All MRM mass spectrometric experiments were performed with a Waters TQD tandem quadrupole detector (Milford, MA) monitored with MassLynx MS software. Samples were analyzed in positive ESI mode with MRM of the transitions *m/z* 492 → *m/z* 116 for Pt(DDTC)_2_, *m/z* 640 → *m/z* 492 for Pt(DDTC)_3_, and *m/z* 355 → *m/z* 116 for the internal standard nickel complex Ni(DDTC)_2_. The capillary voltage, cone voltage, extractor voltage, and RF lens voltage were set at 2.8kV, 25 V, 3 V, and 0.1 V, respectively. The desolvation nitrogen gas flow rate was 650 L/h, cone nitrogen gas flow rate 50 L/h, the source temperature was 150 °C, and the desolvation temperature was held at 350 °C. Samples were separated with an Acquity BEH C18 column (1.7 μm, 2.1 mm i.d. × 150 mm or 50 mm, Waters). The isocratic elution was performed with a mobile phase B ratio of 80% with a flow rate of 0.4 mL/min (A = 0.1% formic acid in water; B = 0.1% formic acid in ACN).

**Supplemental Figure 1.**


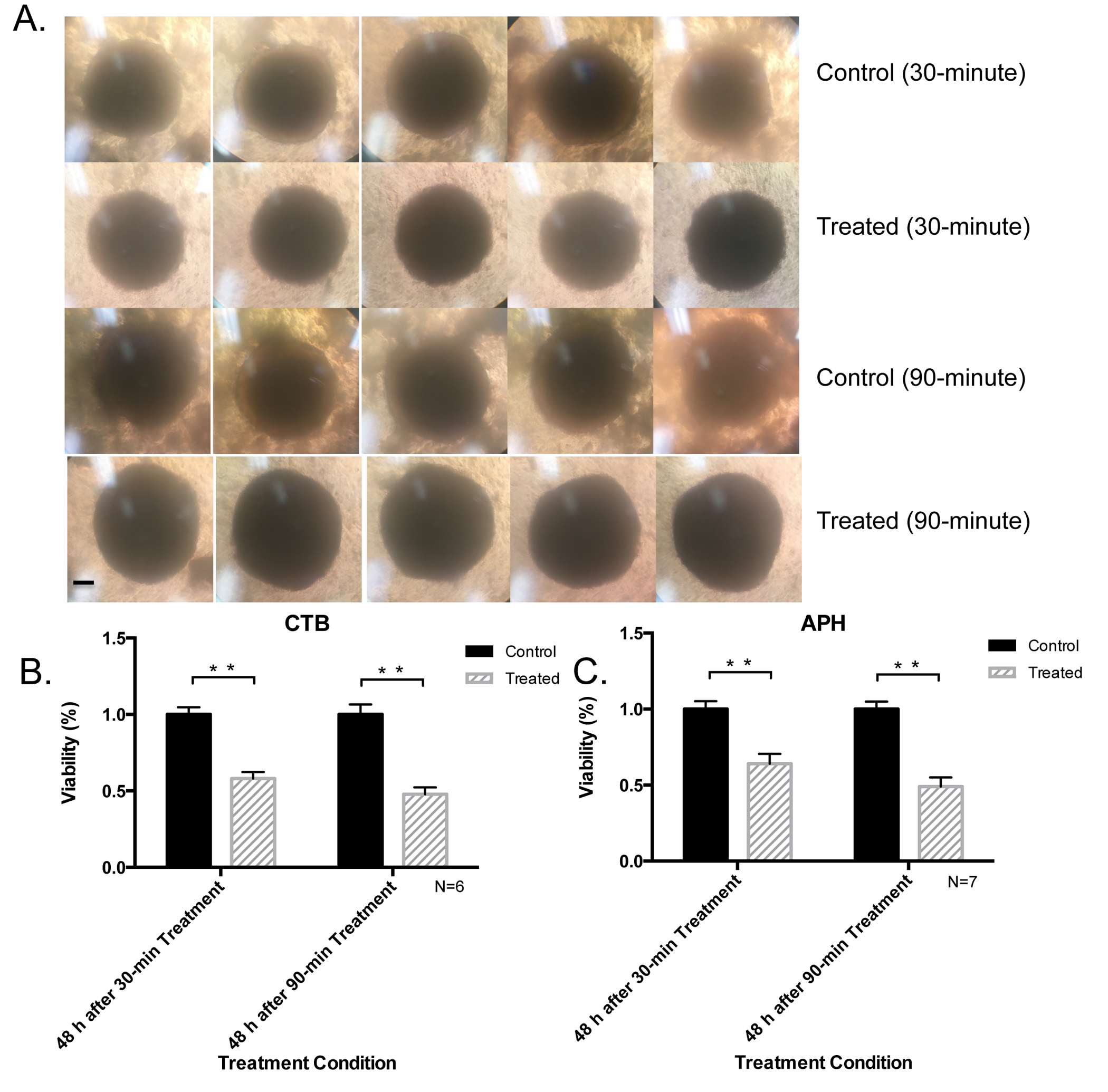


**Supplemental Figure 1.** Microscopic images (A) and Viability of MCTS following HIPEC-like oxaliplatin treatment using Cell Titer-Blue Assay (B) and Acid Phosphatase Assay (C). Scale bar, 200 µm. Error bars, standard deviation. ***p*<0.01

**Supplemental Figure 2**.


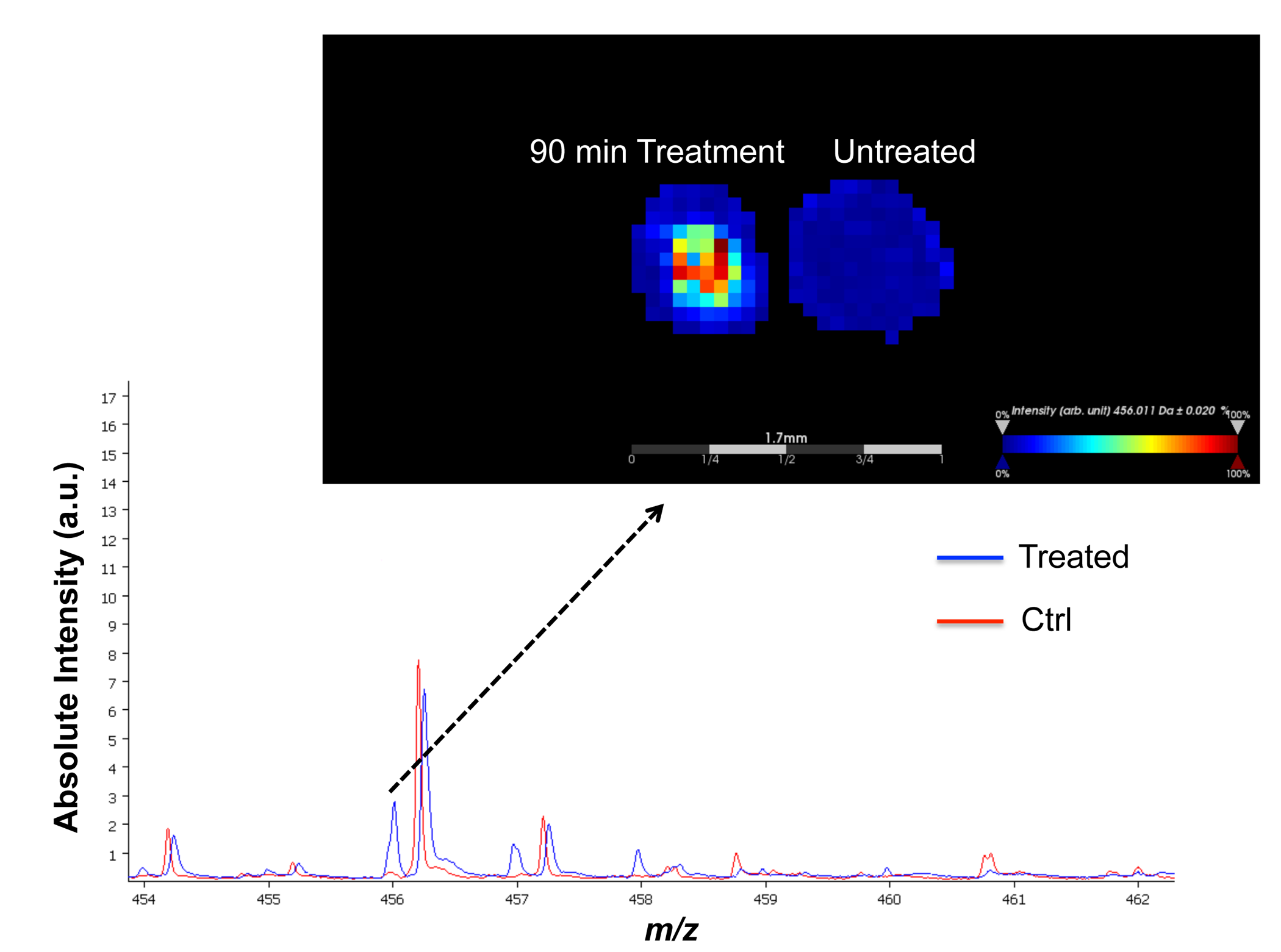


**Supplemental Figure 2.** MALDI IMS results of 90 min treated and untreated MCTS without DDTC derivatization. [Pt(DACHCcCH)(Met)]^+^was detected in drug treated MCTS. Besides the [Pt(DACH)(Met)]^+^, neither the drug nor any other metabolite of oxaliplatin was detected.

**Supplemental Figure 3.**


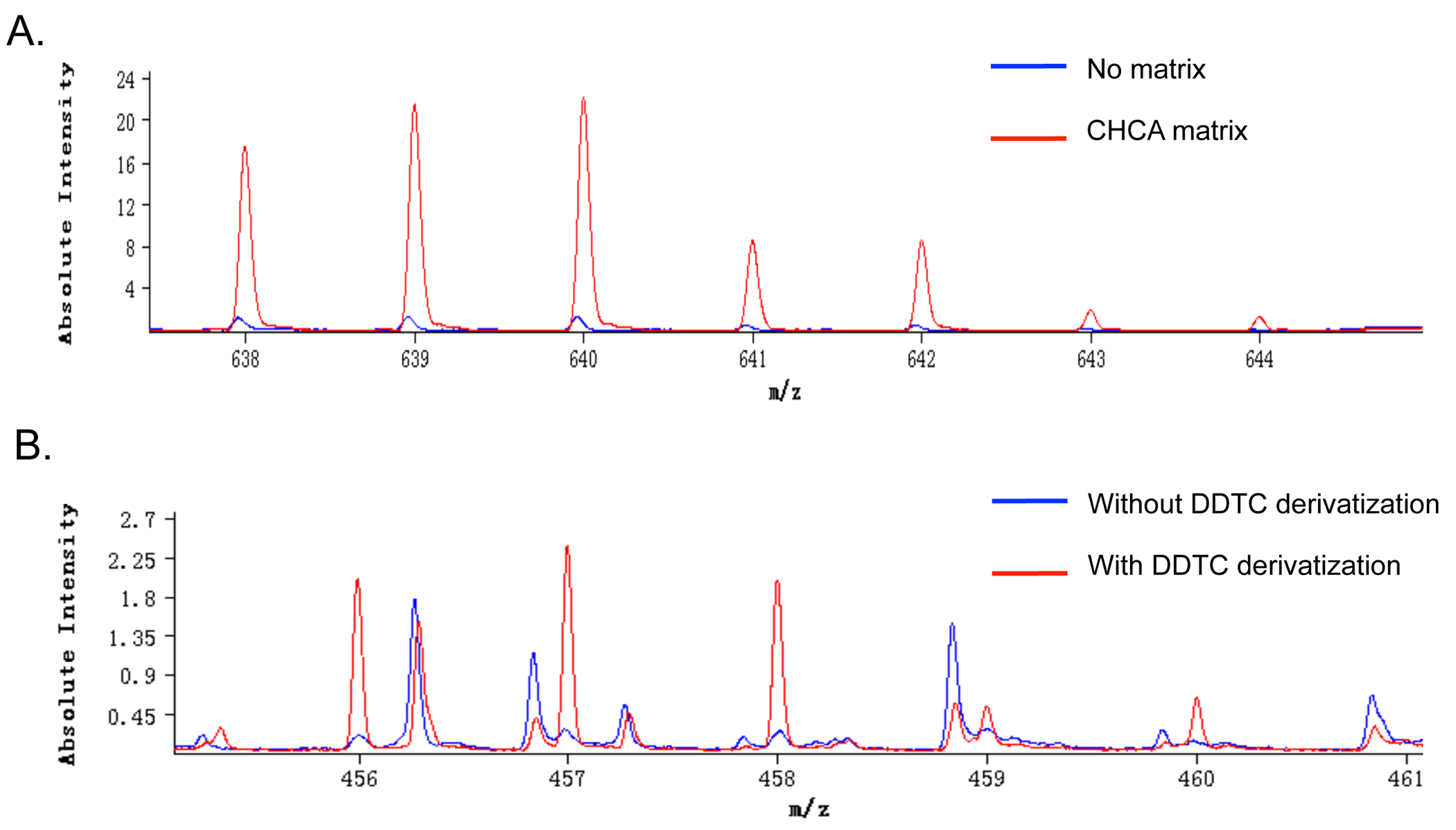


**Supplemental Figure 3.** MALDI spectra acquired from 90 min oxaliplatin treated MCTS. (A) Comparison of peaks of [Pt(DDTC)_3_]^+^ with and without matrix application. (B) Comparison of peaks of [Pt(DACHCcCH)(Met)]^+^ with and without DDTC derivatization.

**Supplemental Figure 4.**


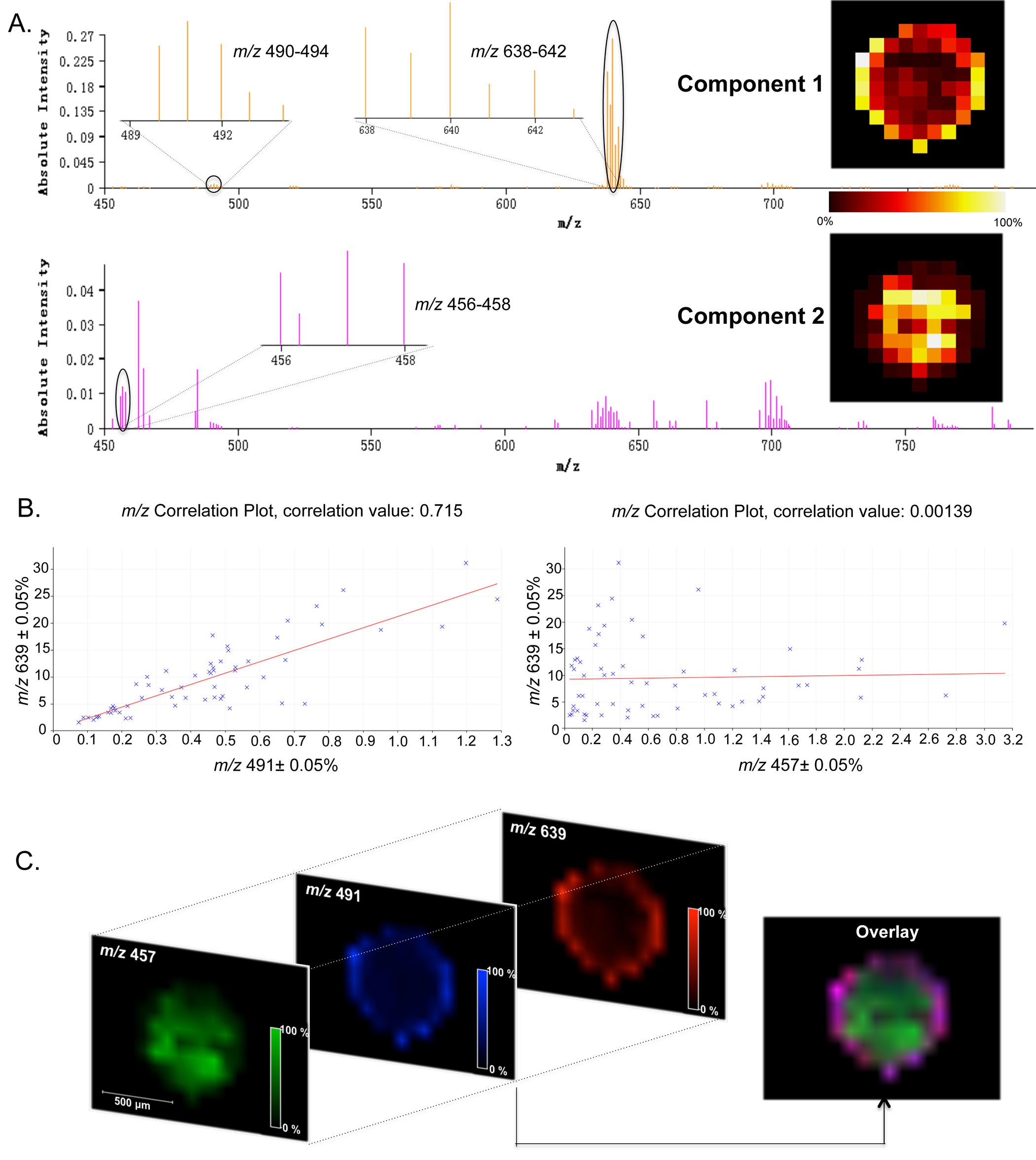


**Supplemental Figure 4.** Statistical analysis to evaluate different localization of Pt species in 90-min HIPEC treated MCTS. (A) pLSA score plots and loading plots showing distinct distribution patterns of Component 1 and Component 2. (B) Evaluation of co-localized and anti-colocalized *m/z* values using correlation analysis. (C) MALDI IMS images of different distributions of Pt species.

**Supplemental Figure 5.**


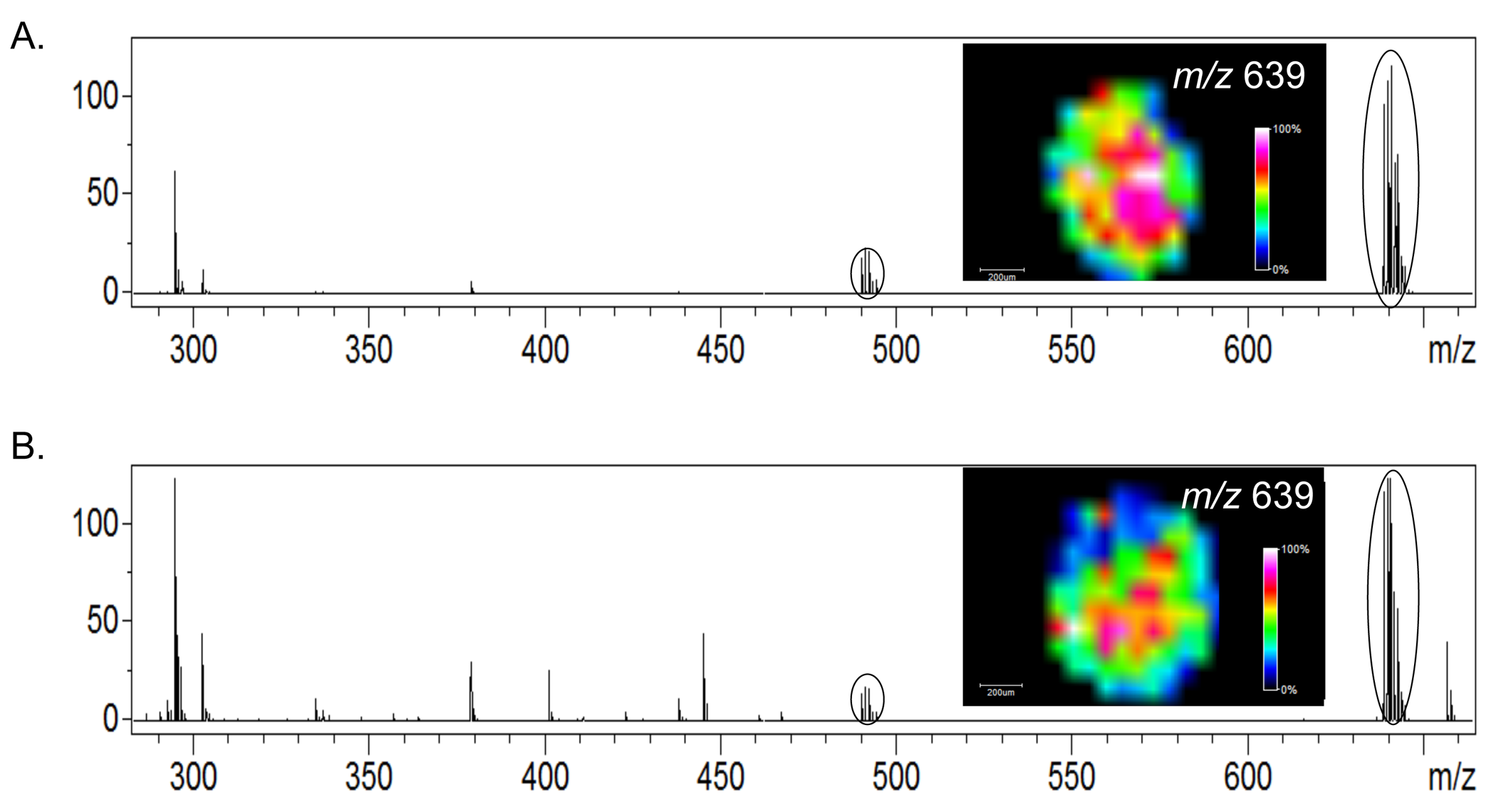


**Supplemental Figure 5.** MALDI IMS results with DDTC derivatization of (A) cisplatin (40 μM, 48 h) or (B) carboplatin (40 μM, 48 h) treated MCTS.

**Supplemental Figure 6.**


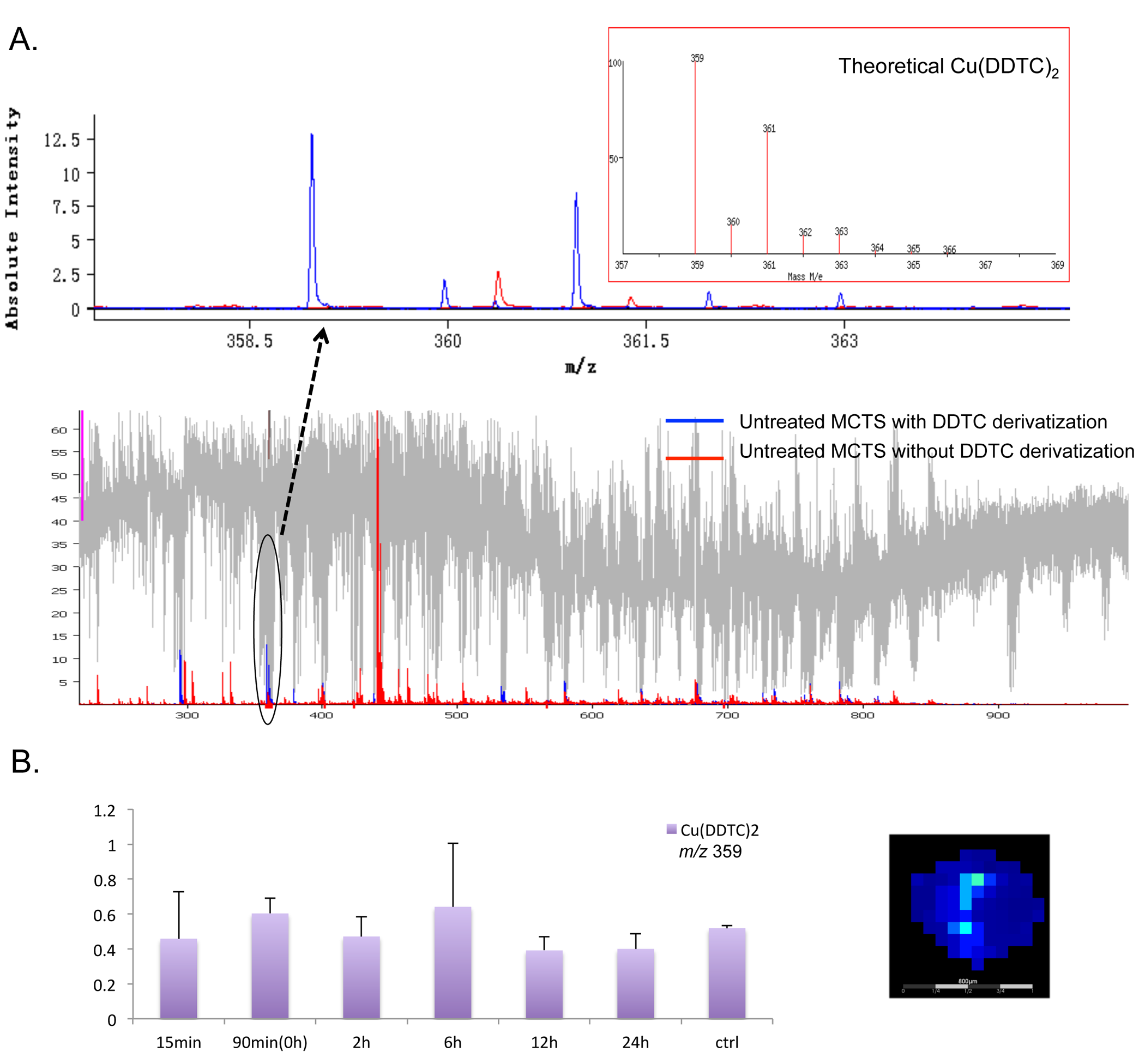


**Supplemental Figure 6.** Detection of Cu(DDTC)_2_ by MALDI IMS. (A) Spectra from untreated MCTS with and without DDTC derivatization. (B) Average signal intensities of [Cu(DDTC)_2_]^+^ in MCTS at different time points, and a typical ion image of its distribution.

**Supplemental Figure 7.**


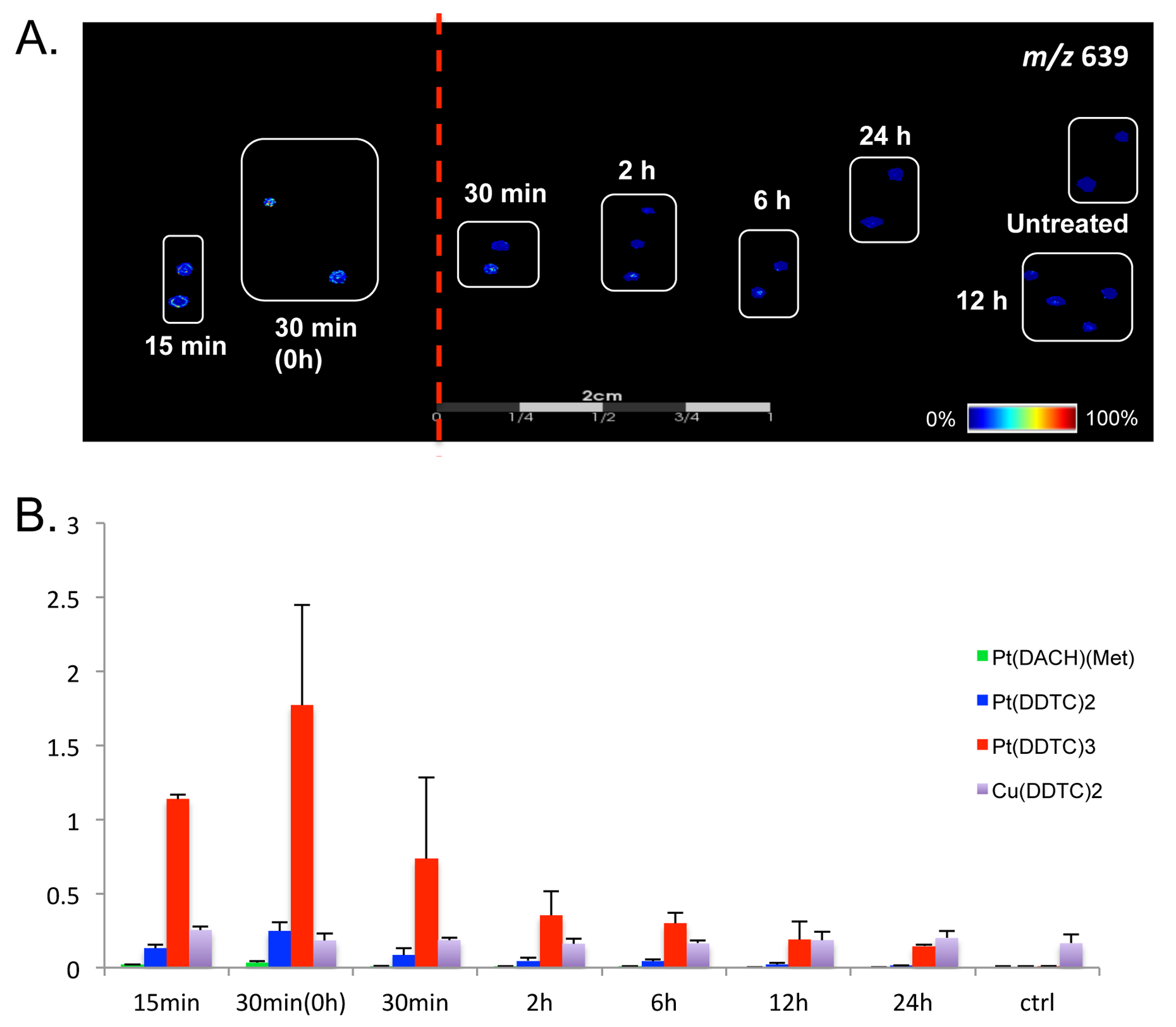


**Supplemental Figure 7.** MALDI IMS results of 30 min HIPEC treated MCTS. (A) Distribution of Pt(DDTC)_3_ at different time points of treatment.(B) Average signal intensities of Pt-related species and Cu(DDTC)_2_.

**Supplemental Figure 8**.


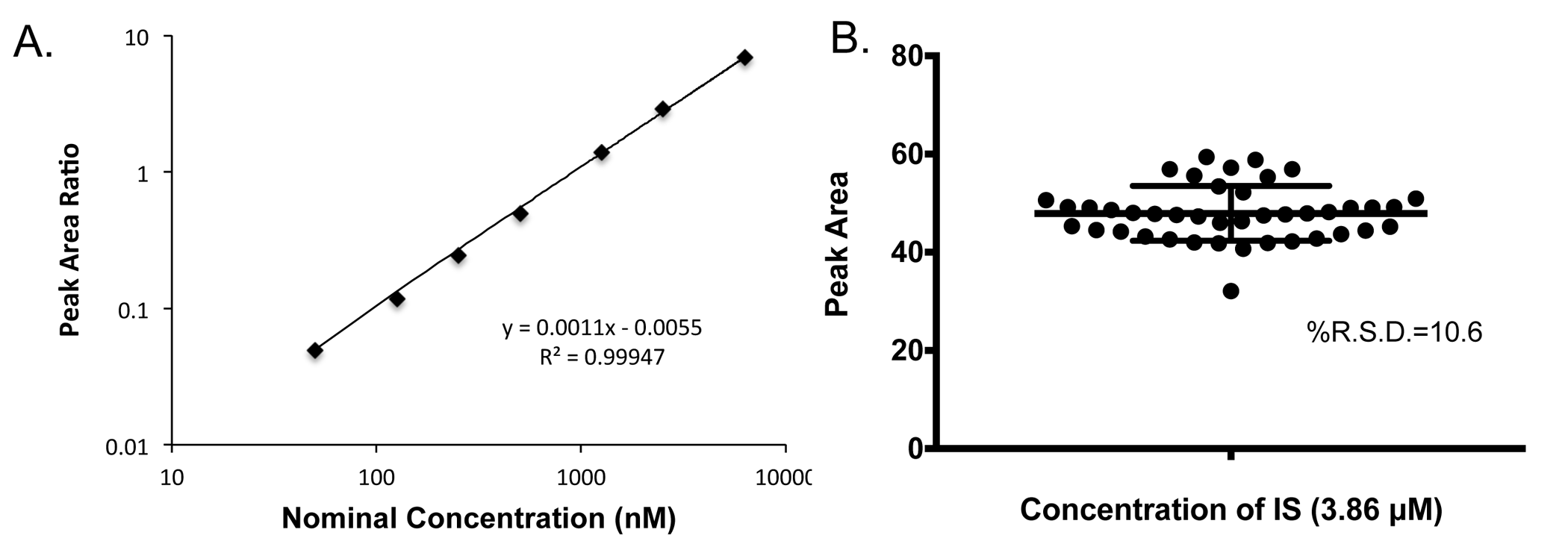


**Supplemental Figure 8.** (A) Representative standard curve of Pt(DDTC)_2_ and Pt(DDTC)_3_. (B) Reproducibility of the internal standard (n=42).


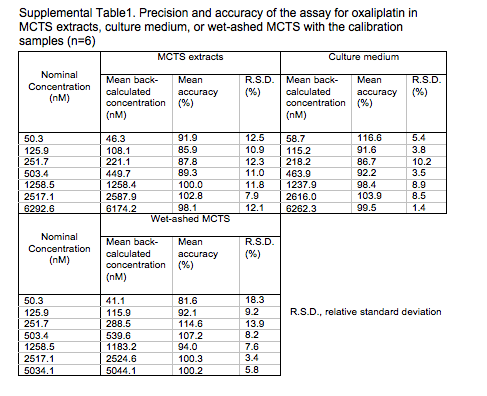


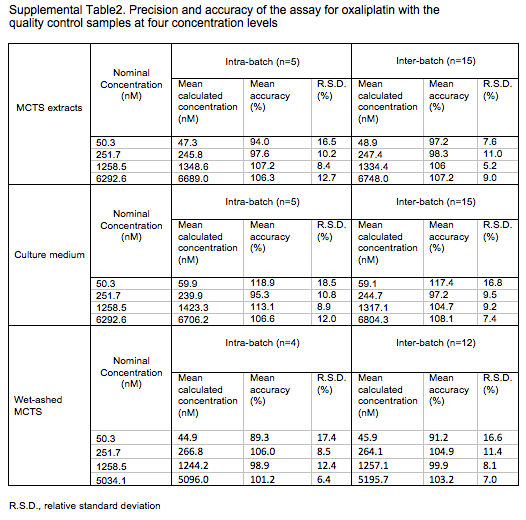


**Supplemental Figure 9.**

**Supplemental Figure 9.** Matrix effect with or without adding the internal standard. Error bars, standard deviation.

**Supplemental Figure10.**


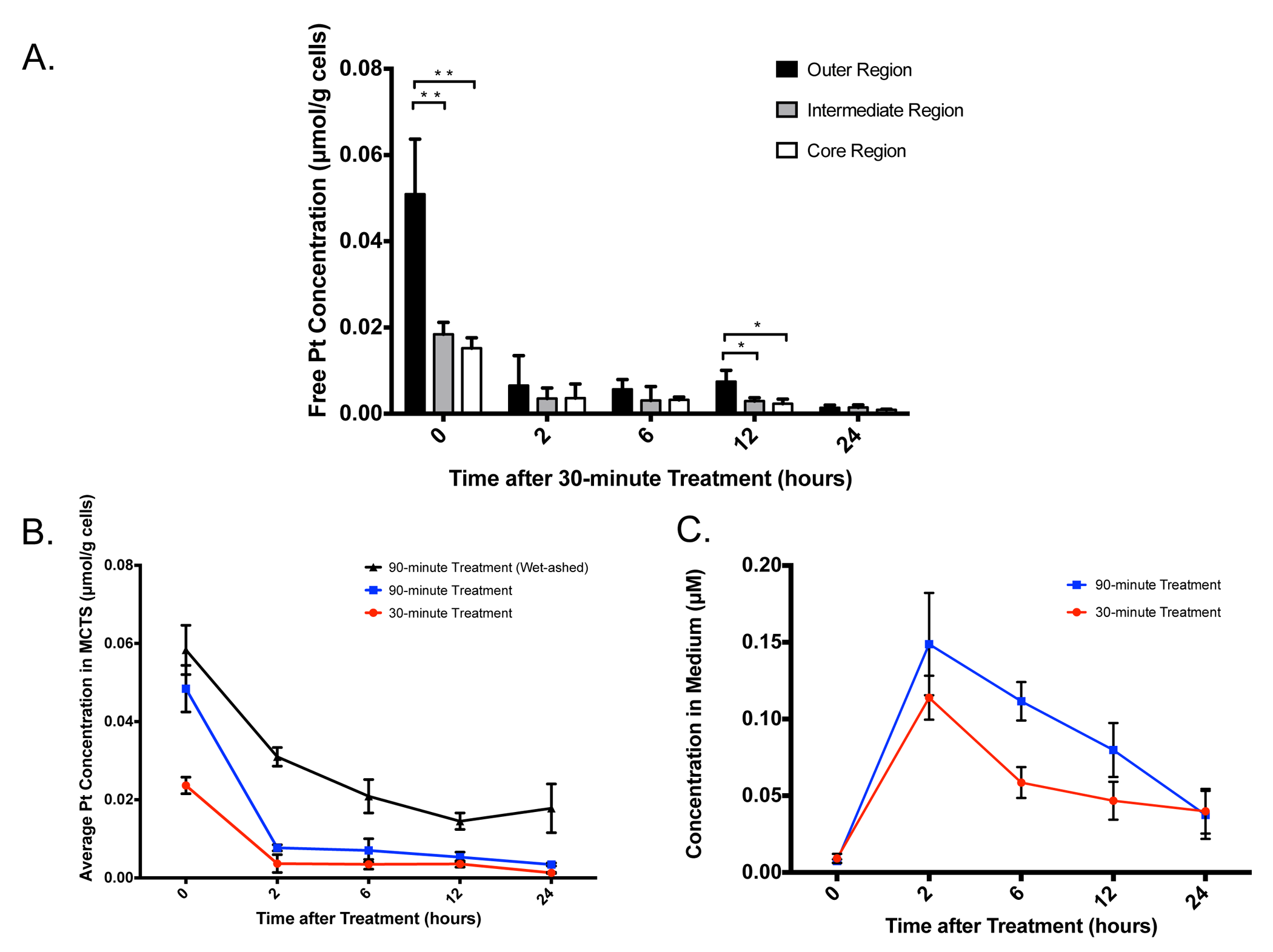


**Supplemental Figure 10.** Quantification of free Pt or total Pt molecules in MCTS. (A) Free drug clearance for 30-min treated MCTS. (B) Average Pt concentration in whole MCTS following HIPEC-like treatment. (C) Quantification of free Pt molecules in culture medium. Error bars, standard deviation. **p*<0.05, ***p*<0.01.

**Supplemental Figure 11**.


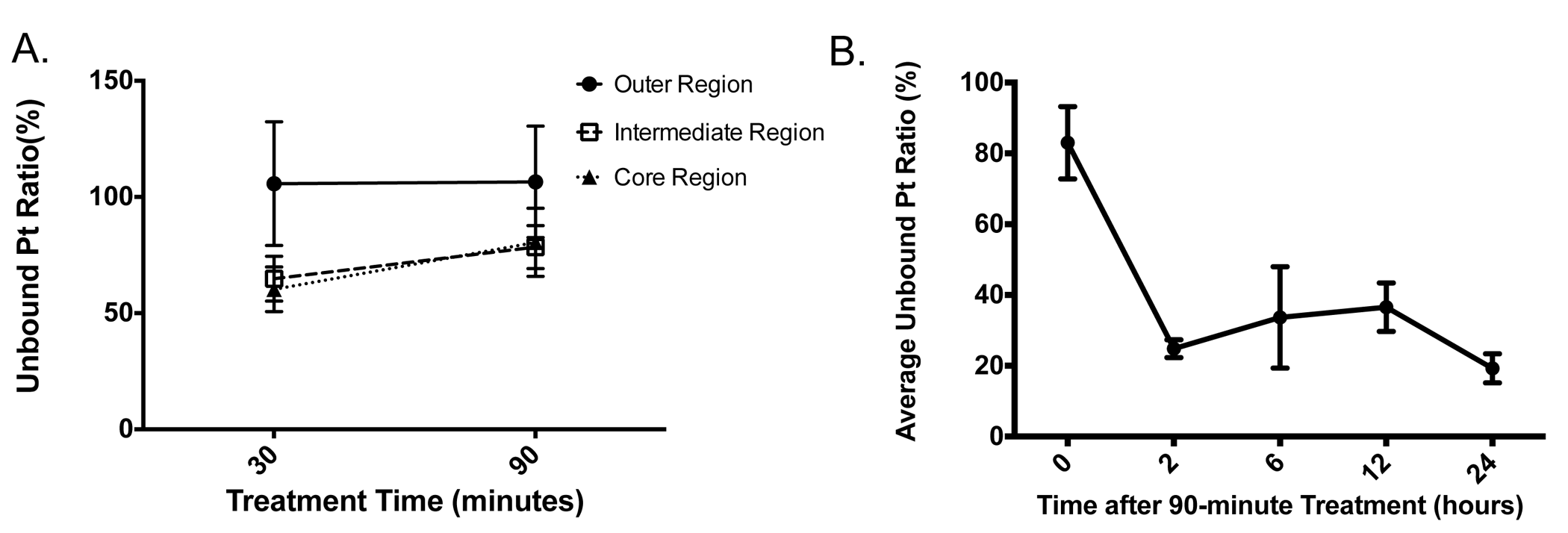


**Supplemental Figure 11.** Unbound Pt ratio in 30-min or 90-min treated MCTS (A). Average unbound Pt ratio following 90-min treated whole MCTS (B). Error bars, standard deviation.
